# The Impact of Chemical Modifications on the Interferon-Inducing and Antiproliferative Activity of Short Double-Stranded Immunostimulating RNA

**DOI:** 10.3390/molecules29133225

**Published:** 2024-07-07

**Authors:** Ali Bishani, Mariya I. Meschaninova, Marina A. Zenkova, Elena L. Chernolovskaya

**Affiliations:** Institute of Chemical Biology and Fundamental Medicine SB RAS, Lavrentiev Ave. 8, 630090 Novosibirsk, Russia; ali1bishani@gmail.com (A.B.); mesch@niboch.nsc.ru (M.I.M.); marzen@niboch.nsc.ru (M.A.Z.)

**Keywords:** immunostimulating RNA, short double-stranded RNA, chemical modifications, cytokine-inducing activity, antitumor activity, IFN alpha

## Abstract

A short 19 bp dsRNA with 3′-trinucleotide overhangs acting as immunostimulating RNA (isRNA) demonstrated strong antiproliferative action against cancer cells, immunostimulatory activity through activation of cytokines and Type-I IFN secretion, as well as anti-tumor and anti-metastatic effects in vivo. The aim of this study was to determine the tolerance of chemical modifications (2′-F, 2′-OMe, PS, cholesterol, and amino acids) located at different positions within this isRNA to its ability to activate the innate immune system. The obtained duplexes were tested in vivo for their ability to activate the synthesis of interferon-α in mice, and in tumor cell cultures for their ability to inhibit their proliferation. The obtained data show that chemical modifications in the composition of isRNA have different effects on its individual functions, including interferon-inducing and antiproliferative effects. The effect of modifications depends not only on the type of modification but also on its location and the surrounding context of the modifications. This study made it possible to identify leader patterns of modifications that enhance the properties of isRNA: F2/F2 and F2_S/F2 for interferon-inducing activity, as well as F2_S5/F2_S5, F2-NH2/F2-NH2, and Ch-F2/Ch-F2 for antiproliferative action. These modifications can improve the pharmacokinetic and pharmacodynamic properties, as well as increase the specificity of isRNA action to obtain the desired effect.

## 1. Introduction

Immunostimulating RNA (isRNA) refers to a class of RNA molecules designed to activate the innate immune system [1]. Unlike traditional RNA-based therapies (such as siRNA, ASOs, antagomirs, and aptamers), which primarily target specific RNAs or proteins [2,3], isRNA aims to stimulate immune responses. It can be used for various therapeutic purposes, including cancer immunotherapy, the treatment of infectious diseases, and autoimmune disorders [4,5]. The immunostimulating effect can be either specific, developing in response to mRNA vaccines encoding a specific antigen [6], or nonspecific, acting through the activation of the innate immune system [7]. isRNA activates the innate immune system by interacting with pattern recognition receptors (PRRs) [4,8]. These receptors recognize specific RNA motifs and trigger immune responses. The immunostimulatory RNA’s location, structure, sequence, and modifications determine which particular PAMPs are involved in recognition. Upon recognition by PRRs, isRNA induces the production of cytokines, such as interferons and interleukins [9]. These cytokines play a crucial role in immune activation and antiviral defense [10,11,12].

RNA is inherently unstable due to endo/exoribonucleases present in body fluids such as the blood serum [13], which can cleave the RNA molecule at different positions [14]. In order to protect isRNA, researchers have been trying extensively to develop protective formulations, such as lipid nanoparticles, for its delivery [15]. Another approach to increase isRNA nuclease resistance is based on the chemical modification of its ribose-phosphate backbone [16]. This approach has been successfully applied for siRNA, and it is used in RNA-based drugs approved for clinical use [17]. Its main task was to increase stability to enzymatic degradation while maintaining biological activity [18]. Unfortunately, little is known about the effects of chemical modifications on the properties of immunostimulatory RNAs: most research efforts have been aimed at blocking the immunostimulatory properties of therapeutic RNAs, such as siRNAs and mRNA vaccines [19]. The main types of modifications used in siRNA include modifications of the 2′-position of ribose, which is involved in RNA cleavage via the trans-esterification mechanism used by most endoribonucleases [19]. Internucleotide phosphate and terminal modifications are used to protect siRNA against exonuclease degradation and can also be used to facilitate the cellular entry of the molecules [20,21].

2′-Fluoro (2′-F) and 2′-*O*-methyl (2′-OMe) modifications are widely used as modifying groups to protect the 2-position of ribose [22]. The 2′-F modification of the ribose residue in siRNA is a well-established approach to enhance its stability and efficacy [23]. The electronegative fluorine atom can mimic the steric and electronic properties of the hydroxyl group, while preventing the action of ribonucleases [24]. However, the number of such modifications in siRNA is limited, in order to eliminate the side effects that may be associated with the accumulation of modified molecules in the cell nucleus [25], using them in positions where other modifications have a detrimental effect on the function [26]. Another option of modification involves the introduction of 2′-OMe groups. This modification occurs in natural RNAs, such as the epitranscriptomic modification of ribosomal RNA (rRNA) [27]. These modifications are strategically aimed at quelling immunostimulatory responses, while preserving the siRNA gene silencing function [28]. Intriguingly, oligoribonucleotides harboring 2′-*O*-methyladenosine residues exhibit the capability to actively antagonize IFN-α secretion induced by other immunostimulatory RNAs [29].

Phosphorothioate (PS) modification is a widely used strategy to enhance the stability of siRNA [30,31]. This chemical modification involves substituting one of the non-bridging oxygen atoms in the phosphate backbone of the RNA with a sulfur atom. The introduction of PS linkages into siRNA can significantly increase its resistance to nuclease degradation in vivo, which is a common challenge for RNA-based therapeutics [32]. PS modifications, when used in large quantities, can cause toxic side effects due to interaction with serum proteins; [33] however, siRNAs with a limited number of PS modifications (two at each end) demonstrate a favorable safety profile [34].

The conjugation of cholesterol to therapeutic RNAs, such as siRNA, is a strategy employed to extend its circulation time in the bloodstream, thereby enhancing the delivery and efficacy of RNA-based therapeutics [35,36]. Cholesterol, a lipophilic molecule, can be conjugated to siRNA to enhance its pharmacokinetic properties, due to the formation of cholesterol complexes with serum lipoproteins [37]. These complexes prevent the rapid renal clearance of siRNA and prolong its presence in the bloodstream [37]. Furthermore, the lipophilic nature of cholesterol facilitates the incorporation of siRNA into lipid membranes, facilitating its cellular uptake [38,39]. Consequently, cholesterol-modified siRNAs exhibit improved biodistribution profiles, enabling the more efficient targeting of tissues and cells [37].

The introduction of an amino linker of different lengths at the 3′- or 5′-end of an oligonucleotide is routinely used for the introduction of labels or ligands. The amino linker itself allows it to protect the oligonucleotide from degradation under the action of exoribonucleases. This approach is actively used to protect oligodeoxyribonucleotides. In the case of ribonucleotides, the main route of their degradation is cleavage under the action of endoribonucleases; however, exoribonucleases also contribute to this process.

Earlier, our group described a short 19 bp dsRNA with 3′-trinucleotide overhangs (immunostimulatory RNA, isRNA) that demonstrated strong antiproliferative action against cancer cells and immunostimulatory activity through the activation of cytokine production (Type-I IFNs) [40]. This duplex was selected among control siRNAs obtained by in vitro transcription due to the terminal transferase activity of T7 RNA polymerase. A significant proportion of the molecules were one nucleotide longer than those provided by the template and had pronounced antiproliferative properties. The chemical synthesis of possible variants made it possible to establish the sequence of a duplex with immunostimulating properties [41]. Since this isRNA lacks significant similarity with human or mouse mRNAs and is one nucleotide longer than canonical siRNAs, it cannot alter the pattern of gene expression through RNA interference. In vitro and in vivo investigations were conducted on the immunostimulatory, interferon-inducing, antiproliferative, anticancer, and antiviral properties of isRNA [9]. As we previously demonstrated, the immunostimulatory effect of this isRNA becomes apparent only when delivery mechanisms are employed, which is consistent with the isRNA’s antiproliferative action, involving the intracellular molecular targets RIG-I and PKR.

The purpose of this study was to determine the tolerance of chemical modifications (F, OMe, PS, cholesterol, amino) located at different positions within this isRNA on its ability to activate the innate immune system. The obtained duplexes were tested in vivo for their ability to activate the synthesis of interferon-α in mice, and in tumor cell cultures for their ability to inhibit their proliferation.

## 2. Results

### 2.1. isRNA Duplexes and Experimental Design

Numerous prior studies have demonstrated the efficacy of nucleotide analogs featuring chemical alterations at the 2′-position of the ribose ring in enhancing the stability of siRNA against nucleases. However, the extensive modification of siRNA has been shown to potentially compromise its functional efficacy [28]. Hence, a systematic exploration of the type, number, and position of modifications in isRNA strands becomes imperative. The following modifications are usually used in siRNA: 2′-F and 2′-OMe, PS modifications, attachment of an amino group and cholesterol at the 5′- and 3′-ends (Table 1). The selected 2′-F and 2′-OMe modification patterns are based on preferential modification CpA, UpA, UpG sites, cleaved by endoribonucleases similarly to the algorithm of selective modification developed by us for siRNA [42]. These identified sites were subjected to modification by 2′-F in the F1 pattern, which contained seven modifications in two strands. The imposition of this pattern on our previous data shows that some modifications are located in sites in which nucleotide substitutions affect the immunostimulating properties; therefore, in the patterns M (with 2′-OMe) and F2 (with 2′-F), the modifications in these places were removed, and these patterns contained four modifications in both strands (Table 1).

The phosphorothioate (PS) modification of oligonucleotides is pivotal for enhancing their pharmacological attributes. The modification of two internucleotide phosphates at both ends of the siRNA strands has been successfully used to protect them from the action of exonucleases. Although oligonucleotides harboring PS modification demonstrate heightened protein-binding affinities, their influence on the interaction of oligoribonucleotides with PRRs is not clear, so we synthetized strands containing 2 PS units at both ends simultaneously (S), at only 3′- or 5′-termini (S3 and S5, respectively), or in the middle (SM) of the strands (Table 1).

The non-modified form of isRNA typically possesses a molecular weight that falls below the renal filtration threshold (30~50 kDa), resulting in rapid clearance from the bloodstream and consequently short in vivo half-lives, thereby limiting their therapeutic potential. Previously, our research team addressed the delivery challenges associated with isRNA. We employed cationic liposomes with diverse modifications to surmount these hurdles and demonstrated that the addition of PEG-containing lipoconjugates to the liposome formulation, which increase the circulation time of the cargo isRNA in the bloodstream, enhances its interferon-inducing activity [9]. To circumvent renal clearance and enhance stability against exonucleases, various strategies have been explored, including the conjugation of bulky moieties such as cholesterol to the 5′- or 3′-termini of RNA strands (Ch- and -Ch, respectively). Herein, we investigate the impact of these terminal modifications on the activity of isRNA and attached cholesterol on the 3′- or 5′-end of the strands via a hexamethylen linker. Cholesterol is a fairly bulky hydrophobic modification and can influence biological effects through different mechanisms; therefore, to assess the tolerance of terminal modifications, we used a small modification—an amino group attached through a similar linker (-NH2), which, however, can contribute to the protection against exonucleases (Table 1). Modification patterns containing PS, Ch, and NH2 modifications were overlaid on the F2 or unmodified N backbone; duplexes containing strand 1 and strand 2 with the same pattern of modifications or with different patterns were obtained to study the biological activity (Table 1).

**Figure 1:**
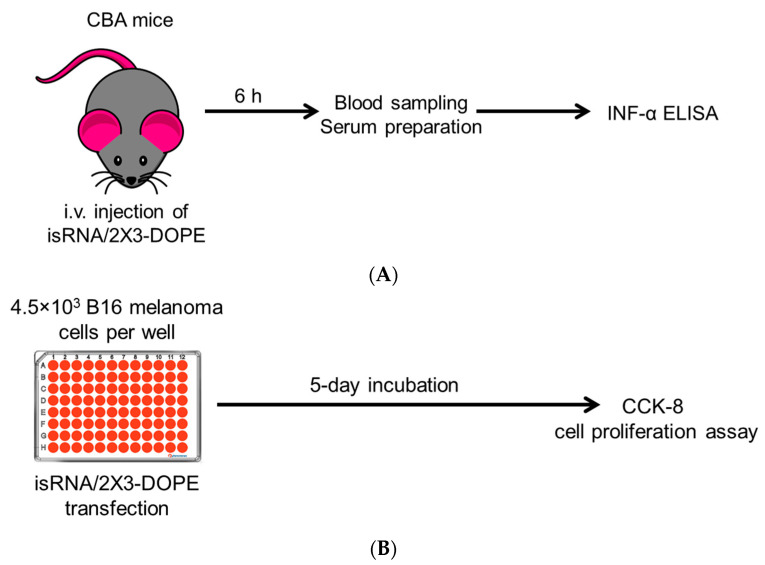
Schemes of experiments. (**A**) Interferon-inducing activity was assayed in vivo: groups of CBA mice (*n* = 3) were i.v. injected with 10 mcg of chemically modified isRNA complexed with 2X3-DOPE at a N/P ratio of 6/1 in 200 μL of OptiMEM. After 6 h, blood was collected, and serum was prepared and used for IFN-α ELISA analysis. (**B**) Antiproliferative activity was assayed in vitro: 4.5 × 10^3^ B16 cells were plated in a 96-well plate and transfected with 100 nM isRNA complexed with 2X3-DOPE at a N/P ratio of 6/1, and 5 days post-transfection, the numbers of cells were determined using the CCK-8 cell proliferation assay.

Immunostimulatory RNA (isRNA) exhibited its activity as an inducer of Type-I interferons, specifically interferon α (IFN-α), and also as an agent with significant antiproliferative and anti-tumor activity. To evaluate the impact of the aforementioned modifications on the biological activities of isRNA, we examined separately their impact on the interferon-inducing activity in CBA mice and the antiproliferative activity in B16 melanoma cell culture (Figure 1). Previously, we showed that for isRNA to exhibit biological activity, it is necessary to ensure its intracellular delivery to target cells; therefore, in both cases, we used pre-complexation of isRNA with cationic liposomes 2X3-DOPE [43], which are highly effective both in vitro and in vivo and act in the presence of serum.

### 2.2. The Impact of Chemical Modifications on the Interferon-Inducing Activity of isRNA

#### 2.2.1. Effect of Terminal Modifications and Ribose Modifications on the Interferon-Inducing Activity of isRNA

Cohorts of CBA mice (n = 3) were subjected to intravenous administration of modified isRNA pre-complexed with 2X3-DOPE to ascertain the impact of various modifications of isRNA on the levels of IFN-α. Subsequent to a six-hour interval post-injection, blood specimens were collected, and the ensuing serum samples were harvested for the ELISA analysis, facilitating a comparative assessment of IFN-α levels across distinct experimental cohorts (Figure 1A).

**Figure 2:**
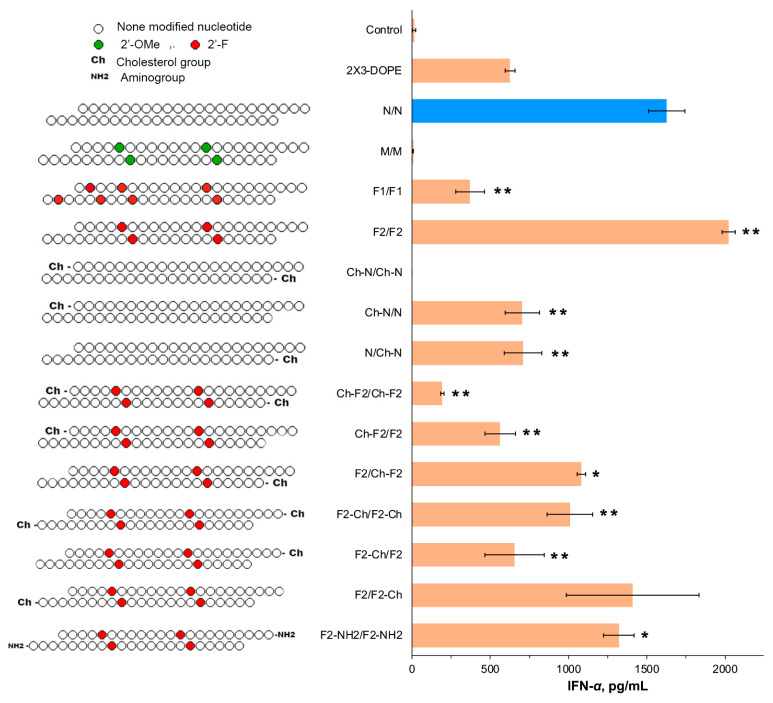
The effect of the terminal and 2′-ribose modifications of isRNA on IFN-α production in CBA mice (*n* = 3) 6 h after i.v. administration of isRNA/liposome complexes. Serum IFN-α levels were measured by ELISA. Untreated control mice. The data represent mean ± standard deviation (SD) calculated from three independent experiments measured in duplicates. Statistically significant differences between experimental groups and the N/N group are indicated by asterisks (* *p* < 0.005; ** *p* < 0.001); ordinary two-way ANOVA, Dunnett’s multiple comparisons test.

The structural alterations introduced at the 2′ position of the ribose moiety within isRNA backbone demonstrated their notable influence on IFN-α synthesis in murine blood upon administration. The unmodified isRNA N/N caused a significant increase in the IFN-α level to 1627 ± 117 pg/mL. Seven 2′-F modifications in F1/F1 elicited an apparent reduction in the effect: a weak elevation in IFN-α to 218 ± 16 pg/mL was detected (Figure 2). In stark contrast, the reduction in the number of 2′-F modifications to four in F2/F2 emerged as the most potent activation, evoking a significant surge in the IFN-α levels up to 2022 ± 41 pg/mL, surpassing the levels elicited by non-modified isRNA. Notably, the inclusion of the 2′-OMe modifications in the same positions (M/M) completely eliminated the interferon-inducing activity. Thus, we chose duplexes with N and F2 patterns to study the influence of terminal modifications.

The introduction of cholesterol at the 5′-termini of both strands within the non-modified isRNA duplex (Ch-N/Ch-N) completely blocked the interferon-inducing activity. Intriguingly, the introduction of the cholesterol group to either one of the strands (the Ch-N/N and N/Ch-N duplexes) led to a roughly twofold reduction in the secreted levels of IFN-α, compared to those induced by the unmodified N/N, as depicted in Figure 2.

Subsequent examination upon the introduction of the cholesterol group to the F2/F2 pattern revealed that the modifications of the 5′-end of both strands (Ch-F2/Ch-F2) failed to elicit a significant augmentation in the IFN-α levels (193 ± 11 pg/mL), whereas the modification at the 3′-end of both strands (F2-Ch/F2-Ch) was less intense than that for F2/F2, but still yielded a significant increase in level up to 1009 ± 145 pg/mL. In the case of isRNAs, wherein the cholesterol group was attached to only one strand demonstrated that Ch attachment to the 5′- or 3′-end of the first strand (Ch-F2/F2 and F2-Ch/F2) elicited a moderate rise in the IFN-α levels to 563 ± 98 and 654 ± 189 pg/mL, respectively. Conversely, the cholesterol modifications of the second strand (F2/Ch-F2 and F2/F2-Ch) were better tolerated and yielded a substantial increase (approximately 1108 ± 145 pg/mL for F2/Ch-F2 and 14,080 ± 423 pg/mL for F2/F2-Ch), as illustrated in Figure 2. Lastly, the introduction of an amine group (-NH2) at the 3′-termini of both strands within the duplex, alongside with the F2 modification (F2-NH2/F2-NH2), elicited a discernible rise in the IFN-α levels (1320 ± 96 pg/mL), as presented in Figure 2.

#### 2.2.2. Effect of Phosphate Modifications on the Interferon-Inducing Activity of isRNA

**Figure 3:**
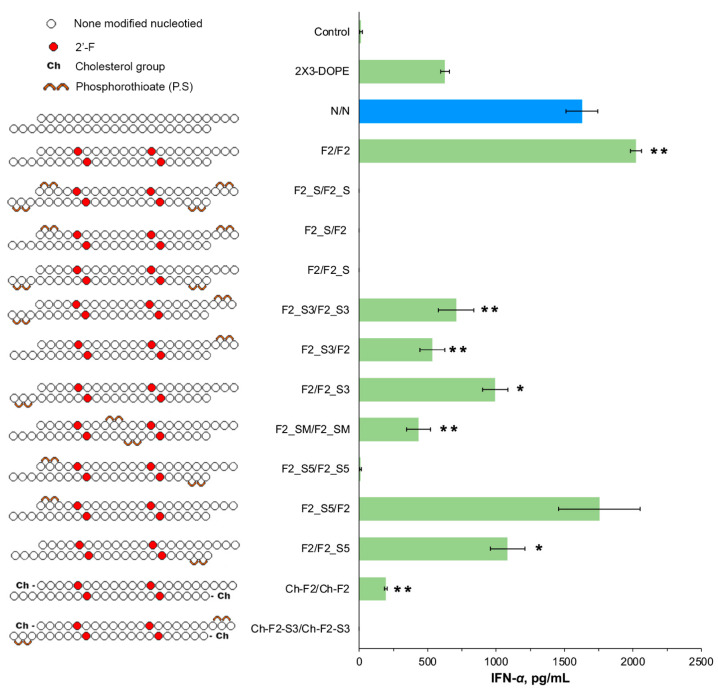
The effect of PS modifications in isRNA on IFN-α production in CBA mice (*n* = 3) 6 h after i.v. administration of isRNA/liposome complexes. Serum IFN-α levels were measured by ELISA. Control-untreated mice. The data represent mean ± standard deviation (SD) calculated from three independent experiments measured in duplicates. Statistically significant differences between experimental groups and the N/N group are indicated by asterisks (* *p* < 0.005; ** *p* < 0.001); ordinary two-way ANOVA, Dunnett’s multiple comparisons test. The data for the modified comparison duplex N/N is shown in blue, the rest in green.

At the next stage, the investigation extended to explore the impact of PS linkages in the F2/F2 backbone on interferon-inducing activity of the respective isRNAs (Figure 3). Integration of two PS groups into the backbone at both termini of both strands (F2_S/F2_S), or of one strand only (F2_S/F2 and F2/F2_S), as well as the 2 PS modifications at the 5′-termini of both strands (F2_S5/F2_S5), resulted in a complete blockade of IFN-α synthesis, as evidenced by the results depicted in Figure 3.

Subsequently, the ramifications of integrating PS at a single position on the strands were examined. The introduction of the modification near the 3′-end of both strands (F2_S3/F2_S3) elicited a moderate response, approximately 707 pg/mL, which was more than twofold less than that observed for F2/F2. Similarly, the same modification applied to the 5′-cholesterol duplex with low activity (Ch-F2_S3/Ch-F2_S3) resulted in a total loss of its interferon-inducing activity. Conversely, heteroduplexes, containing 2 PS modification only at the 3′-end of the second strand (F2/F2_S3) provided higher IFN-α levels (993 ± 92 pg/mL) than those containing similar modifications in the first strand (F2_S3/F2, 533 ± 91 pg/mL). The integration of PS in the middle of the strands in “the mismatch tolerated zone” (F2_SM/F2_SM) resulted in a slight, albeit statistically insignificant, rise in the IFN-α levels (432 ± 87 pg/mL), which is inferior to the efficiency of IFN-α activation under the action of F2_S3 variants, as depicted in Figure 3.

The subsequent introduction of PS modifications at the 5′-termini of the isRNA, concomitant with F2 modification, revealed noteworthy observations. While the introduction of PS modifications to both strands (F2_S5/F2_S5) blocked the IFN-α synthesis, modifications of individual strands did not prevent the induction of the elevated IFN-α levels. Notably, the modification of the first strand only (F2_S5/F2) caused a significant elevation in the IFN-α level, up to 1755 ± 238 pg/mL. Similarly, 5′-terminal PS modifications in the second strand (F2/F2_S5) provided a lower but still significant level of IFN-α (1100 pg/mL), as depicted in Figure 3.

Based on the above findings, it can be deduced that the F2 modification pattern alone (F2/F2) or in combination with PS at the 5′-termini of the first strand (F2_S5/F2) emerges as efficacious, playing a pivotal role in augmenting the interferon-inducing activity of isRNA. Regarding terminal modifications, the integration of cholesterol onto the second strand, alongside the F2 presence (F2/F2-Ch), or the incorporation of -NH2 at the 3′-termini of both strands, exhibits potential in eliciting substantial levels of IFN-α.

### 2.3. The Impact of Chemical Modifications on the Antiproliferative Activity of isRNA

#### 2.3.1. Effect of Terminal Modifications and Ribose Modifications on the Antiproliferative Activity of isRNA

To elucidate the impact of chemical modifications on the antiproliferative efficacy of isRNA, mice melanoma B16 cells were seeded at a density of 4.5 × 10^3^ cells per well and subsequently transfected with chemically modified isRNA complexed with 2X3-DOPE, across three distinct concentrations (25, 50, and 100 nM). These cultures were maintained for 5 days, with daily monitoring to exclude overgrowth. Subsequently, the CCK-8 assay was used to assess the number of living cells, and the IC50 values were used as a parameter estimating the impact of chemical modifications on the cells’ growth rate.

The unmodified isRNA (N/N), as previously determined, elicited the inhibition of cell growth and proliferation, with optimal efficacy observed at a concentration of 100 nM, yielding an IC_50_ value of 68 nM. Notably, modifications introduced at the 2′ position of the ribose moiety within isRNA exerted a profound impact on its activity.

**Figure 4:**
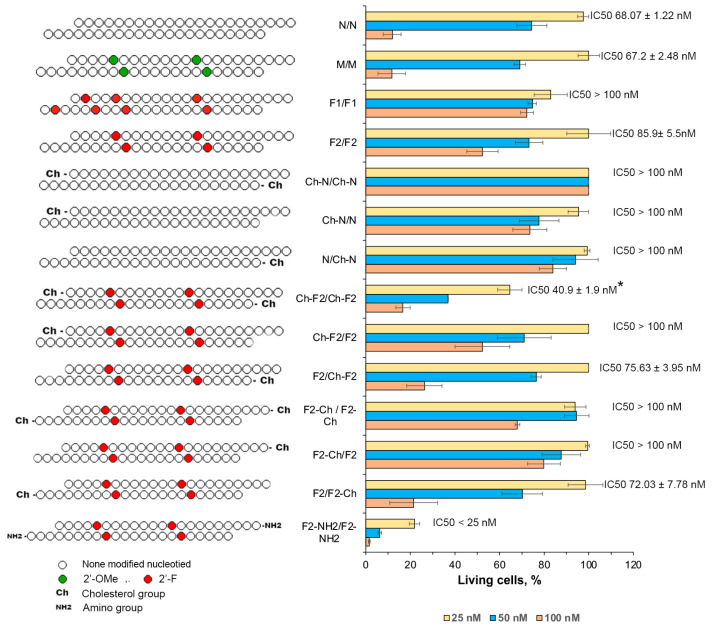
The effect of the terminal and 2′-ribose modifications in isRNA on the antiproliferative activity of isRNA in melanoma B16 cells. The relative viability of cells was evaluated using the CCK-8 assay. The data are shown as mean values ± standard deviations (SD), obtained from tree independent experiments. The number of cells in control (non-treated) was set as 100%. Statistically significant differences between the IC50 values of the experimental and the N/N groups are marked by asterisks (* *p* < 0.05). Statistical analysis was conducted employing an ordinary two-way ANOVA, followed by Dunnett’s multiple comparisons test.

The 2′-F-containing duplexes F1/F1 (seven modifications) and F2/F2 (four modifications) failed to elicit a pronounced effect on cell proliferation, even at the highest concentration, and did not reach 50% inhibition of cell growth (IC_50_ > 100 nM). In contrast to the effect on interferon-inducing activity, the 2′-OMe-containing duplex (M/M) induced marked inhibition, with efficiency comparable to that of the unmodified duplex, with an IC_50_ value of 67.2 nM (Figure 4).

The introduction of a cholesterol moiety to the 5′-termini of the N/N duplexes resulted in a substantial inhibition in the case of cholesterol attached to one strand or a total loss of activity in the case of cholesterols attached to both strands (Figure 4). The cholesterol modifications on the 5′-end had a completely different effect on the duplexes with the F2 pattern: retention of activity for Ch-F2/F2, moderate activation for F2/Ch-F2, and significant activation of the antiproliferative effect for the duplex with cholesterol on both chains Ch-F2/Ch-F2 (IC50 40.9 nM), which exceeds the activity of the unmodified and the 2′-OMe-modified duplexes. The cholesterol modification at the 3′-termini of both strands of the F2 patterns inhibited its antiproliferative activity, which decreased in the order F2/F2-Ch (IC50 72 nM) > F2-Ch/F2-Ch (IC50 > 100 nM)> F2-Ch/F2 (IC50 > 100 nM) (Figure 4).

The most efficient modification entailed the introduction of an amine group at the 3′-termini of both strands in combination with the F2 pattern (F2-NH2/F2-NH2), eliciting robust proliferation inhibition even at the lowest concentration, with an IC_50_ value below 25 nM (Figure 4).

#### 2.3.2. Effect of Phosphate Modifications on the Antiproliferative Activity of isRNA

**Figure 5:**
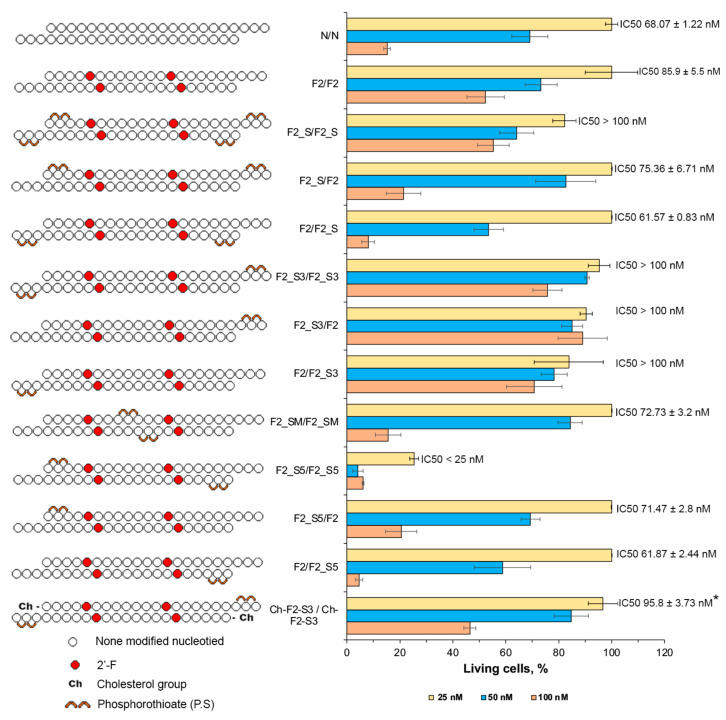
The effect of PS modifications in isRNA on the antiproliferative activity of isRNA in melanoma B16 cells. The relative viability of cells was evaluated using the CCK-8 assay. The data are shown as mean values ± standard deviations (SD), obtained from tree independent experiments. The number of cells in control (non-treated) was set as 100%. Statistically significant differences between the IC50 values of experimental and the N/N group are marked by asterisks (* *p* < 0.05). Statistical analysis was conducted employing ordinary two-way ANOVA, followed by Dunnett’s multiple comparisons test.

The subsequent focus of the investigation centered on the application of the 2′F2 modification in conjunction with the integration of PS at various positions along the strands. Incorporating 2 PS groups at both ends on both strands (F2_S/F2_S) elicited a strong inhibition of antiproliferative activity. Conversely, the introduction of this modification to the first strand only (F2_S/F2) resulted in a modest inhibition at the highest concentration (IC50 75.3 nM), with a more pronounced effect observed when PS modifications were introduced to the second strand (F2/F2_S, IC50 61.6 nM) (Figure 5).

Introducing PS at the 3′-termini of the duplex, whether on both strands (F2_S3/F2_S3) or on each strand alone (F2_S3/F2, F2/F2_S3), abrogated the antiproliferative effect of isRNA. Conversely, the introduction of the same modification at the 5′-termini exhibited enhanced activity, particularly when applied to both strands (F2_S5/F2_S5), where it exerted a robust impact, inducing significant proliferation inhibition, even at the lowest concentration, with an IC50 value below 25 nM. When the modification was applied to the first strand only (F2_S5/F2), inhibition was observed at the highest concentration (IC50 71.5 nM). A more pronounced effect was noted when PS was introduced to the 5′-termini of the second strand (F2/F2_S5, IC50 61.9 nM), as illustrated in Figure 5. Similarly, the introduction of 2 PS at the middle part on both strands resulted in an IC50 value of 72.7 nM, comparable with non-modified duplex. The combination of the 2′F2 modification with 2 PS at the 3′-termini, in addition to 5′-cholesterol on both ends, did not elicit a significant inhibition at the concentrations employed, and increased the IC50 value from 40.9 to 95.8 nM (Figure 4 and Figure 5).

In summary, the introduction of the 2′-F modification, as opposed to 2′-OMe, failed to enhance its antiproliferative efficacy. However, a notable improvement in functionality was observed upon its conjunction with terminal modifications, such as the inclusion of a cholesterol moiety at the 5′-termini or an amine group at the 3′-termini of both strands. Furthermore, the incorporation of PS at the 5′-end notably augmented its antiproliferative activity. Noteworthy is the observation that the introduction of chemical modifications solely to the second strand resulted in a more pronounced antiproliferative effect compared to modifications on the first strand.

## 3. Discussion

The effects of chemical modifications on the non-specific immunostimulatory properties of therapeutic ribonucleic acids, including small interfering RNA (siRNA) and mRNA vaccines, have been an important focus of extensive investigation. The goal of such studies is to prevent unwanted activation of innate immunity and associated nonspecific effects, increase the specificity of action, and reduce the toxicity of nucleic acid-based drugs. The opposite task—maintaining the immunostimulating activity of immunostimulating RNA and DNA—as a rule remains outside the scope of interest of researchers, despite the fact that immunostimulating nucleic acids are actively being studied as a means for the treatment of tumors and infectious diseases, as well as adjuvants.

Accumulating evidence attests to the ability of unaltered siRNA duplexes to elicit activation of the mammalian innate immune response, culminating in the production of inflammatory cytokines and interferons (IFNs) [44]. This phenomenon is mainly orchestrated by immune cell populations, frequently via Toll-like receptor (TLR) signaling cascades [44,45]. However, even in non-immune cells, such duplexes cause global changes in the gene expression profile, affecting proliferation, transcription, translation, and other cellular functions. A detailed investigation underscored the pivotal role of sequence specificity and structural features in governing the immunogenicity of siRNA [44,46,47], while simultaneously illuminating the promise of chemical modification strategies in mitigating off-target effects. The referenced studies precisely described the intracellular pathways behind innate immune activation by siRNA molecules and delineated the fundamental principles for the rational design of sequences and chemical modifications for the development of safe and efficacious therapeutic modalities that lack nonspecific immunostimulation.

Chemical modifications, including 2′-OMe and 2′-F substitutions, have been demonstrated to exert noticeable effects on the immune activation properties of siRNA. The study [48] revealed that the elicitation of immune responses by siRNA/liposome complexes in murine models transcends mere sequence specificity. Notably, all 12 non-modified siRNA duplexes examined triggered a robust cytokine release, including IFN-α, underscoring the independence of immune activation from sequence context. However, upon exposure to varied permutations of 2′-OMe, 2′-F, and phosphorothioate substitutions, the introduction of three 2′-OMe substitutions exclusively within the sense strand sufficed to attenuate immune activation triggered by the siRNA duplexes. Remarkably, these modifications exhibited diminished efficacy in mitigating the immune response elicited by single-stranded siRNAs. This observation suggests that the chemical alteration of siRNA holds promise as a feasible strategy to alleviate unintentional immune activation in therapeutic siRNAs or, conversely, to potentiate immune activation in the context of immunostimulatory RNA. The investigation suggested that Toll-like receptor 3 (TLR3)-signaling is not involved in the immune stimulation by siRNA/liposome complexes, resulting in the robust immune activation observed in TLR3 knockout mice upon administration of siRNAs [48]. In the realm of mRNA vaccine development, mitigating the non-specific immune response poses a pivotal challenge, necessitating intricate chemical modifications. Significantly, tailored modifications to the mRNA molecule hold promise for supporting its stability while simultaneously diminishing its immunogenicity. A cornerstone in this pursuit involves the strategic incorporation of modified nucleosides, such as pseudouridine or 5-methylcytidine, which have demonstrated pronounced efficacy in blocking the innate immune response to mRNA, thereby enhancing its translational efficiency [49].

In a previous investigation undertaken by our research group [41], an extensive analysis of the sequence-dependent immunostimulatory properties of isRNA under the present study was conducted both in vivo and in vitro. This comprehensive inquiry involved the deliberate introduction of mismatches and insertions at various positions along the nucleotide strands, with the aim of elucidating the regions that impeded activity, as well as those that tolerated alterations. Sensitive and insensitive to nucleotide substitutions, duplex regions have been identified for the antiproliferative effects of isRNA. Based on these findings, the present study endeavors to extend these findings by strategically introducing chemical modifications at the identified loci, with the overarching objective of delineating both deleterious and optimal positions, conducive to enhancing functionality.

Consistent with precedent findings documented in the scientific literature, our observations corroborate that 2′-ribose modifications, including 2′-OMe and 2′-F modifications, could impede immune responses. The obtained data demonstrated that the effect of these modifications on the inhibition of immune activation is position-specific: the inhibitory effect is exerted by modifications in positions that belong to the “substitution-sensitive” zone, while modifications in “substitution-insensitive” zones are well tolerated. Our data revealed that the F2 modification pattern demonstrated marked efficacy, positioning it as an optimal candidate for subsequent exploration, particularly in conjunction with other modifications (Figure 2 and Figure 4).

**Figure 6:**
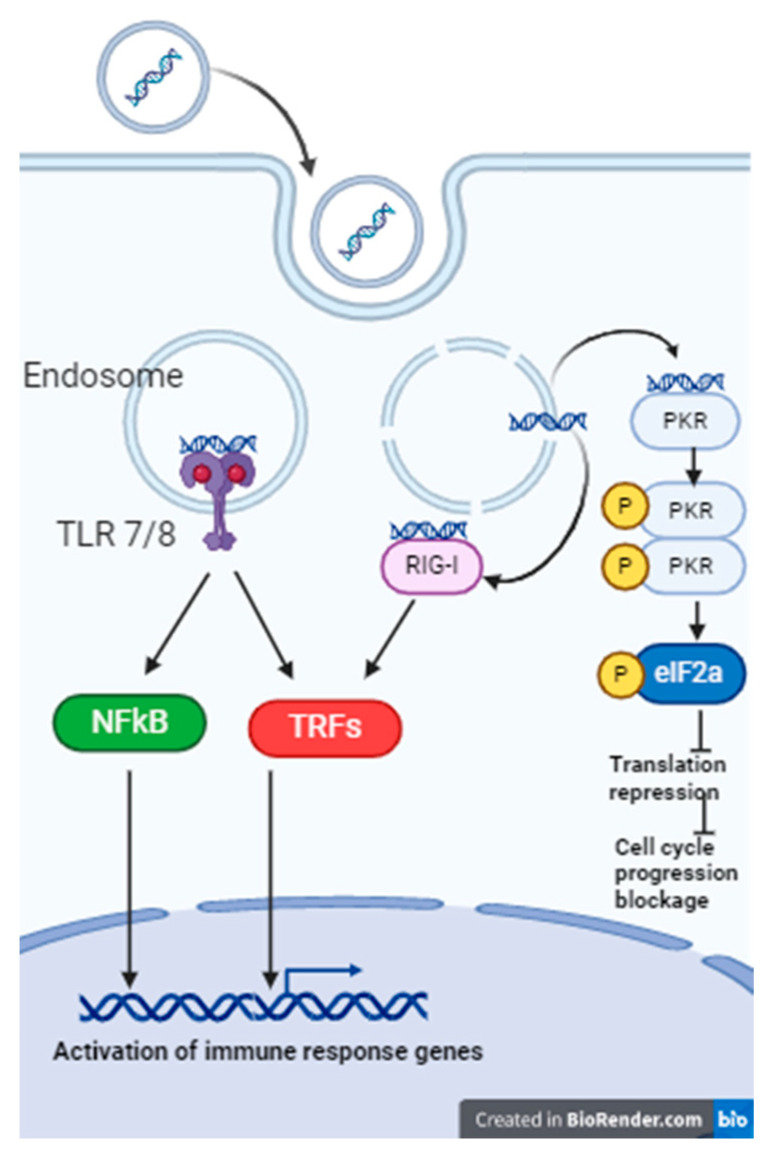
The putative mechanism of activation of the innate immune system by isRNA. dsRNA of different sequences and lengths can activate different PRRs. Presumably, isRNA can bind to TLR 7/8, RIG-I, and/or PKR receptors, which can result in the production of proinflammatory and anti-inflammatory cytokines or in blocking the cell cycle progression. Each receptor can activate a specific pathway by activating and binding different adapter proteins. Recognition of isRNA by more than one receptor may explain differences in the effects of chemical modifications on individual biological functions.

An important result of the present study is the decoupling between interferon-inducing and antiproliferative activities exhibited by chemically modified isRNA, reflecting different in vivo and in vitro behaviors, as defined by our findings. The data obtained suggest that these functions are carried out independently, through the activation of different signaling pathways after recognition of isRNA by different PRRs; therefore, the sensitivity of different regions of the duplex to modifications differs for these activities (Figure 2 and Figure 4). The putative mechanism of activation of the innate immune system under the influence of isRNA is presented in Figure 6.

The introduction of phosphorothioate modifications significantly reduces or even blocks the immunostimulating activities of isRNA, especially when both strands are modified at both ends. PS modifications at the midpoint of duplex strands or on a 3′ terminus exhibited tolerance, without impeding the activity of isRNA. Such duplexes with PS modifications elicited IFN-α induction, accompanied by a modest anti-proliferative impact against B16 tumor cells, although it is less potent compared to the unaltered N/N or F2/F2.

Previous investigations underlined the detrimental impact of mismatches at the 3′ terminus on isRNA functionality [41]. Our current study extends these observations, revealing a pronounced influence of bulky chemical alterations proximal to the 3′ terminus on isRNA function. This underscores the pivotal role of this position in isRNA recognition by binding proteins or cellular receptors, thereby instigating immune responses. Notably, the introduction of a cholesterol moiety exclusively at the 3′-termini of both isRNA strands resulted in the complete blockade of antiproliferative function and the reduction of interferon synthesis, whereas unilateral introduction diminished efficacy by half.

In our preliminary experiments [50], it was previously shown that the attachment of NH2 to the 5′-end of strand 1 or 2 or the attachment of cholesterol to the 5′-end of strand 1 does not block interferon-inducing activity. However, the study used Lipofectamine 2000 as the delivery vehicle, and the levels of interferon α were very low, so these data cannot be directly compared.

The combination of the F2 pattern with cholesterol incorporation at the 5′-end of both strands notably attenuated IFN-α synthesis, with a reduced impact when limited to the first strand and a negligible effect when restricted to the second strand. Conversely, incorporation of the same modification on both strands significantly enhanced antiproliferative function, with augmented efficacy noted upon selective integration into the second strand. Likewise, the combination of F2 with PS near the 5′-termini of both strands resulted in a blockade of IFN-α induction, whereas the unilateral integration demonstrated a less pronounced impact. The in vitro analyses corroborated these trends, revealing a potent inhibition of tumor cell growth upon integration of these modifications into both strands, with enhanced efficacy observed upon selective integration into the second strand (Figure 3 and Figure 5).

In the study conducted by Kabilova et al. [41], it was elucidated that substitution of the terminal nucleotide at the 3′-end of nucleotide strands exhibited a discernible tolerance solely on the first strand. Moreover, the addition of several nucleotides to the 3′-ends was found to impede biological activity, whereas the addition of a single nucleotide was deemed permissible within the experimental framework. Analogously, in the scope of our investigation, the application of the F2 pattern, in conjunction with either cholesterol or phosphorothioate, at the 3′-termini of both strands did not abolish interferon induction; however, it proved to be less efficacious compared to the unmodified N/N or F2/F2. Notably, the introduction of these modifications to the first strand reduced the interferon-inducing activity, whereas their incorporation in the second strand enhanced the interferon-inducing activity.

The augmentation of interferon-inducing activity and the enhancement of the antiproliferative effect observed upon the introduction of the NH2 group at the 3′-termini of both strands, in conjunction with the F2 pattern, are of particular interest. This enhancement may be attributed to the relatively small size of the NH2 group compared to the cholesterol moiety, resulting in better tolerance, in combination with the protection of the duplex from the action of exoribonucleases.

Discriminating the impact of various chemical modifications and their locations on immune responses evoked by RNA therapeutics assumes paramount significance. Here, we have shown that chemical modifications in the composition of isRNA have different effects on its individual functions, such as interferon-inducing and antiproliferative effects. The portability of modifications depends not only on the type of modification but also on its location and the surrounding context of the modifications. This study made it possible to identify key modification patterns that enhance the properties of isRNA: F2/F2 and F2_S/F2 for interferon-inducing activity, as well as F2_S5/F2_S5, F2-NH2/F2-NH2, and Ch-F2/Ch-F2 for antiproliferative action. These modifications can improve the pharmacokinetic and pharmacodynamic properties, as well as increase the specificity of isRNA action to obtain the desired effect.

## 4. Materials and Methods

### 4.1. isRNA Synthesis

Oligoribonucleotides and their analogs (Table 1) were synthesized by the phosphoramidite method on an automatic ASM-800 synthesizer (Biosset, Novosibirsk, Russia). In the synthesis, sulfurizing reagent II, 2′-*O*-TBDMS-protected, 2′-F-, 2′-OMe-ribophosphoramidites, CPG polymeric carriers with an attached first nucleoside, and 3′-PT-amino-modifier C6 CPG (Glen Research, Sterling, VA, USA) were used. 3′-Cholesterol siRNA conjugates were obtained using a cholesterol-modified polymer carrier synthesized by analogy with the method described in [51]. siRNA conjugates containing a cholesterol residue with a hexamethylene linker at the 5′-end were obtained by analogy with the method described in [52]. Schemes and descriptions of the preparation of cholesterol derivatives are given in the Supplementary Materials (Figures S1 and S2). After standard deprotection, the target products were isolated by preparative gel electrophoresis in 15% polyacrylamide gel under denaturing conditions, followed by elution of the products with 0.3 M NaClO_4_. The isolated products were desalted on a Sep-Pac C18 cartridge (Waters, Milford, MA, USA) or Amicon Ultra 3K (Millipore, Burlington, MA, USA) and precipitated with a 2% NaClO_4_ solution in acetone. isRNAs (50 µM) were annealed in a buffer comprising 30 mM HEPES-KOH (pH 7.4), 100 mM sodium acetate, and 2 mM magnesium acetate. Annealing was achieved by heating at 90 °C for 5 min, followed by gradual cooling to room temperature. The formation of the complementary complex was confirmed by native electrophoresis in 15% PAGE in a Tris-Borate-EDTA buffer, pH 8.3. Subsequently, the isRNA preparations were stored at −20 °C until required.

### 4.2. isRNA/liposome Complex Preparation

Complexes containing isRNA and 2X3-DOPE cationic liposomes were prepared in a serum-free OptiMEM medium (Invitrogen, Waltham, MA, USA). Equal volumes of the isRNA solution, with a final concentration of 3.5 µM, and the liposome solution, with a final concentration of 150 µM, were mixed together. The resulting mixture was then incubated at room temperature for 20 min. Subsequently, the complexes, containing 10 μg of isRNA per mouse, were intravenously injected into mice in 200 μL of OptiMEM. The nucleic acid/liposome complexes were pre-formed at N/P ratios of 6/1. Particle size and zeta potential were measured using a Zetasizer Nano ZS (Malvern Panalytical Ltd., Malvern, UK). The average hydrodynamic diameter (nm) was obtained from particle number distributions; the measurements were repeated three times (Table S1).

### 4.3. Mice

Female adult CBA mice (20–24 g) were purchased from the Center for Genetic Resources of Laboratory Animals at the Institute of Cytology and Genetics SB RAS. The mice were housed in the vivarium of the Institute of Chemical Biology and Fundamental Medicine, SB RAS, under natural light conditions and provided with a standard laboratory diet (GOST (State Standard) R 5025892) [53], in accordance with the international guidelines outlined in the European Convention for the Protection of Vertebrate Animals Used for Experimental Studies (1997). All experimental procedures adhered to the regulations stipulated by the Russian State Standards (R 51000.3-96 and 51000.4-96) [54,55] for laboratory practice in preclinical studies. Ethical approval for the experimental protocols was obtained from the Committee on the Ethics of Animal Experiments at the Institute of Cytology and Genetics of SB RAS (protocol No. 51 from 23 May 2019).

### 4.4. ELISA Analysis of IFN-α Levels in Murine Blood Serum

In each experiment, CBA mice (n = 3) were intravenously administered with either 10 µg (750 nmol) of isRNA pre-complexed with 47.25 nmol of 2X3-DOPE at an N/P ratio of 6/1, or with liposomes alone, both dissolved in 200 µL of sterile OptiMEM medium. After 6 h, blood samples were collected from the retroorbital sinus and allowed to clot at 37 °C for 30 min to obtain serum. Subsequent centrifugation was performed to separate the serum. The levels of IFN-α were quantified using the mouse interferon alpha 2 matched antibody pair kit (Elabscience, Houston, TX, USA), following the manufacturer’s protocol. Experiments were repeated three times. Each serum sample was analyzed in duplicate.

### 4.5. Tumor Cell Line

B16 melanoma cells were obtained from the N. N. Blokhin Cancer Research Center in Moscow, Russia. The cells were cultured in Dulbecco’s Modified Eagle Medium (DMEM) supplemented with 10% fetal bovine serum (FBS) and 1% antibiotic–antimycotic solution. Cultures were maintained in a humidified environment with 5% CO_2_/95% air at 37 °C, with regular passaging to sustain exponential growth.

### 4.6. Antiproliferative Activity

Melanoma B16 cells were plated in 96-well flat-bottom plates at a density of 4.5 × 10^3^ cells per well in antibiotic-free medium one day prior to transfection. The cells were then transfected in triplicate with varying concentrations (25, 50, and 100 nM) of isRNA pre-complexed with 2X3-DOPE liposomes, or with liposomes alone, and incubated at 37 °C. Five days post-transfection, 10 μL of a 0.5 mg/mL solution of cell-counting kit CCK-8 (Nanjing Vazyme Biotech, Nanjing, China) was added to each well of the plate, followed by a 2 h incubation at 37 °C in a CO_2_ incubator. Then, the absorbance was measured spectrophotometrically at 450 and 620 nm using a Multiscan RC instrument (Labsystems, Vantaa, Finland).

### 4.7. Ethical Guidelines

The handling of animals strictly adhered to the guidelines outlined in the ECC Directive 2010/63/EU [56], ensuring their proper care and use in laboratory settings. Approval for the protocol was granted by the Committee on the Ethics of Animal Experiments under the jurisdiction of the Administration of the Siberian Branch of the Russian Academy of Sciences.

### 4.8. Statistical Analysis

Statistical analysis was conducted using GraphPad Prism 9.5.1 (GraphPad Software, Inc., San Diego, CA, USA) and Microsoft Excel 2019 (Microsoft Corporation, Redmond, WA, USA). Data are presented as mean ± standard deviation (SD). Statistically significant differences were assessed utilizing an ordinary two-way ANOVA coupled with Dunnett’s multiple comparisons test. A significance threshold of *p* < 0.05 was applied to determine differences.

## Figures and Tables

**Table 1:** Sequences of isRNA and its modified analogs.

Designation	Sequence ^1^	
	First Strand	Second Strand
N	GUGUCAGGCUUUCAGAUUUUUU	AAAUCUGAAAGCCUGACACUUU
M	GUGUCmAGGCUUUCmAGAUUUUUU	AAAUCUmGAAAGCCUmGACACUUU
F1	GUfGUCfAGGCUUUCfAGAUUUUUU	AAAUCUfGAAAGCCUfGACfACUUfA
F2	GUGUCfAGGCUUUCfAGAUUUUUU	AAAUCUfGAAAGCCUfGACACUUU
F2_S	GsUsGUCfAGGCUUUCfAGAUUUUsUsU	AsAsAUCUfGAAAGCCUfGACACUsUsU
F2_S3	GUGUCfAGGCUUUCfAGAUUUUsUsU	AAAUCUfGAAAGCCUfGACACUsUsU
F2_S5	GsUsGUCfAGGCUUUCfAGAUUUUUU	AsAsAUCUfGAAAGCCUfGACACUUU
F2_SM	GUGUCfAGGCUsUsUCfAGAUUUUUU	AAAUCUfGAsAsAGCCUfGACACUUU
Ch-F2	Ch-GUGUCfAGGCUUUCfAGAUUUUUU	Ch-AAAUCUfGAAAGCCUfGACACUUU
Ch-F2_S3	Ch-GUGUCfAGGCUUUCfAGAUUUUsUsU	Ch-AAAUCUfGAAAGCCUfGACACUsUsU
F2-NH2	GUGUCfAGGCUUUCfAGAUUUUUU-NH2	AAAUCUfGAAAGCCUfGACACUUU-NH2
F2-Ch	GUGUCfAGGCUUUCfAGAUUUUUU-Ch	AAAUCUfGAAAGCCUfGACACUUU-Ch
Ch-N	Ch-GUGUCAGGCUUUCAGAUUUUUU	Ch-AAAUCUGAAAGCCUGACACUUU

^1^ Sequences are presented in the 5′–3′ direction: Cm, Um—2′-*O*-methyl modified nucleotides; Cf, Uf—2′-fluoro modified nucleotides; s—phosphorothioate modification; Ch–cholesterol residue attached to the 5′- or 3′-end of the first and second strand via a hexamethylenediamine or serinol linker, respectively; NH2—hexamethyleneamine. The designations of the strands contain indications of the composition of the modifications included in them; N, M, F1, and F2 indicate, respectively, non-modified, 2′-OMe, and 2′-F (patterns F1 and F2); the 5′-terminal modifications are indicated to the left of the designation (Ch-); the 3-terminal modifications are indicated to the right (-Ch, -NH2); modifications of the phosphate group are indicated through the underscore and indicate their 5′-(S5), 3′-(S3), and middle (SM) locations. In the following, duplexes are given as 1 strand/2 strand.

## Data Availability

The data presented in this study are available in this article.

## Supplementary Materials

The following supporting information can be downloaded at: https://www.mdpi.com/article/10.3390/molecules29133225/s1, Figure S1: Synthesis of cholesterol-modified CPG; Figure S2: Synthesis of 5′-cholesterol-modified oligonucleotide, Table S1: Hydrodynamic diameters and ζ-potentials of lipoplexes formed by isRNA and 2X3-DOPE liposomes.
