# Supplementary material for: The Impact of Chemical Modifications on the Interferon-Inducing and Antiproliferative Activity of Short Double-Stranded Immunostimulating RNA
Source: Molecules. 2024 Jul 7;29(13):3225. doi: 10.3390/molecules29133225 (PMC11243415; doi:10.3390/molecules29133225)
Supplement: Supplementary file 1 [file molecules-29-03225-s001.zip › molecules-3056943-supplementary.pdf]

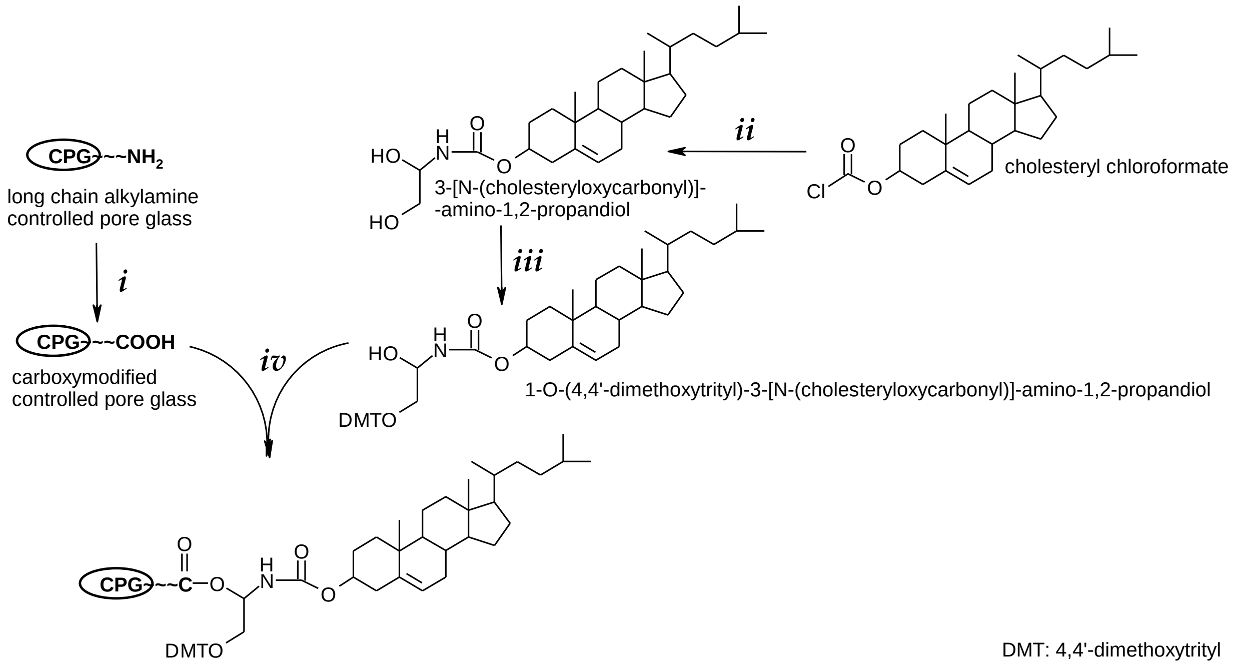

Figure S1. Synthesis of cholesterol-modified CPG: ***i***) long chain alkylamine controlled pore glass (1 g (0.2 mmol/g), Cat#27791, Sigma-Aldrich), succinic anhydride (5 mmol, Cat#239690, Sigma-Aldrich), 1-methylimidazole (5 mmol, Cat#8.05852, Sigma-Aldrich), pyridine (5 ml, Cat#270970, Sigma-Aldrich), 12 h, 25°C; ***ii***) cholesteryl chloroformate (1 mmol, Cat#C77007, Sigma-Aldrich), trimethylamine (1 mmol, Cat#90340, Sigma-Aldrich), 3-amino-1,2-propandiol (1 mmol, Cat#A76001, Sigma-Aldrich), dichloromethane (5 ml, Cat#1.06044, Supelco), 1 h, 25°C; ***iii***) 3-[*N*-(cholesteryloxycarbonyl)]-amino-1,2-propandiol (1 mmol), 4,4′-dimethoxytrityl chloride (1.1 mmol, Cat#100013, Sigma-Aldrich), pyridine (3 ml, Cat#270970, Sigma-Aldrich), 1 h, 25°C; ***iv***) carboxymodified controlled pore glass (0.1 g), 1-*O*-(4,4′-dimethoxytrityl)-3-[*N*-(cholesteryloxycarbonyl)]-amino-1,2-propandiol (0.04 mmol), 2,4,6-triisopropylbenzenesulfonyl chloride (0.04 mmol, Cat#119490, Sigma-Aldrich), 1-methylimidazole (0.12 mmol, Cat#8.05852, Sigma-Aldrich), dry acetonitrile (1 ml, Cat#1.00030, Supelco), 2 h, 37°C [1, 2].

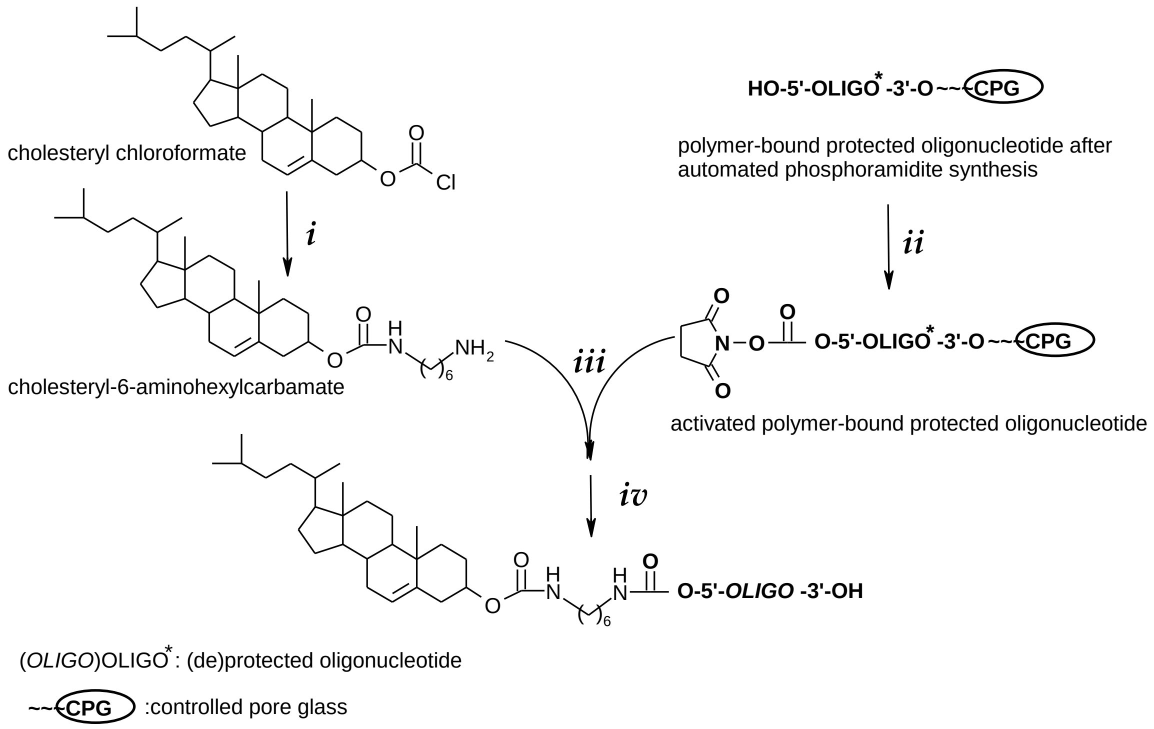

Figure S2. Synthesis of 5′-cholesterol-modified oligonucleotide: ***i***) cholesteryl chloroformate (2 mmol, Cat#C77007, Sigma-Aldrich), triethylamine (2 mmol, Cat#90340, Sigma-Aldrich), 1,6-hexanediamine (10 mmol, Cat#AC120641000, Thermo Scientific Chemicals), dichloromethane (5 ml, Cat#1.06044, Supelco), 1 h, 25°C; ***ii***) polymer-bound protected oligonucleotide after automated phosphoramidite synthesis (10 mg (0.3 μmol)), *N,N′*-disuccinimidyl carbonate (0.1 mmol, Cat#239690, Sigma-Aldrich), pyridine (0.03 ml, Cat#270970, Sigma-Aldrich), dry acetonitrile (0.27 ml, Cat#1.00030, Supelco), 1 h, 37°C; ***iii***) activated polymer-bound protected oligonucleotide (10 mg), cholesteryl-6-aminohexylcarbamate (19 μmol), pyridine (0.04 ml, Cat#270970, Sigma-Aldrich), dichloromethane (0.36 ml, Cat#1.06044, Supelco), 16 h, 37°C; ***iv***) standart deprotection [3].

1. Efimov, V.A., Buryakova, A.A., Reverdatto, S.V., Chakhmakhcheva, O.G. (1983). Use of N-methylimidazole phosphotriester method for the synthesis of oligonucleotides useful in recombinant DNA studies. Bioorg Khim (Russ) 1983, 9, 1367–1381.

2. Chernikov, I.V.; Gladkikh, D.V.; Meschaninova, M.I.; Karelina, U.A.; Ven'yaminova, A.G.; Zenkova, M.A.; Vlassov, V.V.; Chernolovskaya, E.L. Fluorophore Labeling Affects the Cellular Accumulation and Gene Silencing Activity of Cholesterol-Modified siRNAs In Vitro. Nucleic Acid Ther 2019, 29, 33–43, doi:10.1089/nat.2018.0745.

3. Meschaninova, M.I.; Novopashina, D.S.; Semikolenova, O.A.; Silnikov, V.N.; Venyaminova, A.G. Novel Convenient Approach to the Solid-Phase Synthesis of Oligonucleotide Conjugates. Molecules 2019, 24, 4266, doi:10.3390/molecules24234266

Table S1. Hydrodynamic diameters and ζ-potentials of lipoplexes formed by isRNA and 2X3-DOPE liposomes. N/P ratio = 1/6 in all isRNA/liposome complexes.

Lipoplex	**Size, d nm**	**ζ, mV**
—	125.6 ± 0.6	42.5 ± 8.4
N/N	218.8 ± 4.6	24.0 ± 0.3
M/M	219.3 ± 1.2	25.2 ± 1.8
F1/F1	220. ± 3.7	25.8 ± 2.1
F2/F2	219.8 ± 2.2	25.3 ± 1.7
Ch-N/ Ch-N	210.4 ± 3.3	26.0 ± 0.9
Ch-N/N	212.7 ± 2.5	25.6 ± 1.4
N/Ch-N	216.5 ± 4.1	25.5 ± 4.7
Ch-F2/Ch-F2	209.9 ± 5.2	26.1 ± 6.3
Ch-F2/F2	214.4 ± 2.2	25.9 ± 4.0
F2/Ch-F2	210.6 ± 3.9	26.0 ± 3.8
F2-Ch/F2- Ch	220.2 ± 4.7	25.7 ± 2.9
F2-Ch/F2	219.8 ± 5.1	25.2 ± 5.3
F2/F2-Ch	218.9 ± 4.4	25.5 ± 2.0
F2-NH2/F2-NH2	219.6 ± 0.9	24.6 ± 2.2
F2_S/ F2_S	220.8 ± 3.6	26.3 ± 1.5
F2_S/F2	219.8 ± 0.7	25.8 ± 4.3
F2/F2_S	219.6 ± 1.4	25.6 ± 0.5
F2_S3/ F2_S3	219.9 ± 2.7	25.7 ± 6.1
F2_S3/F2	219.5 ± 5.3	25.1 ± 1.1
F2/F2_S3	219.4 ± 4.6	25.4 ± 6.3
F2_SM/ F2_SM	220.5 ± 2.8	24.6 ± 2.5
F2_S5/ F2_S5	219.8 ± 4.4	24.9 ± 5.7
F2_S5/F2	219.0 ± 3.6	25.0 ± 0.6
F2/ F2_S5	219.2 ± 3.9	25.2 ± 1.4
Ch-F2_S3/Ch-F2_S3	221.3 ± 3.7	26.4 ± 1.8
